# Practices and perspectives on dying at home in Norwegian home care services – a secondary analysis of qualitative data

**DOI:** 10.1186/s12904-026-02121-0

**Published:** 2026-05-01

**Authors:** Kristin Valen, Ida Linn Johnsen Enerstvedt, Hilde Håland, Kristin Ådnøy Eriksen, Malin Knutsen Glette

**Affiliations:** 1 Institute of Health and Caring Sciences, Western Norway University of Applied Sciences, Haugesund, Norway; 2 Center for Development of Institutional and Home Care Services (CDIH) in Norway, Haugesund municipality, Haugesund, Norway

**Keywords:** Palliative care, Structured care model, Days at home, Home death, Advance care planning, Peer support

## Abstract

**Background:**

There is increasing recognition that individuals of all ages with life-limiting conditions benefit from palliative care. Despite a preference for dying at home, most patients still spend their final days in hospital. The WHO encourages member states to integrate palliative care across all levels of their health system.

**Aim:**

This study aims to explore how home care services in a Norwegian municipality implement practices that enable patients to die at home, and how healthcare professionals experience these practices.

**Methods:**

A secondary analysis was conducted using qualitative data originally collected in May 2024 for the evaluation of a structured care model for days at home and home death. The dataset comprised four focus groups, one with members of the project team who had developed the model, one with home care unit managers, and two with healthcare workers involved in applying the structured care model in practice. The secondary analysis was conducted between May and August 2025, using Braun and Clarke’s thematic analysis.

**Results:**

The analyses resulted in the following three themes, which describe how home care services implement practices enabling patients to die at home, as well as their experiences of this practice: T1) Advancing palliative care through focused projects: enhancing competence and addressing challenges; T2) Spending the final days at home: identifying patients with palliative care needs and providing compassionate and effective care, and, T3) Sharing responsibility versus reducing the workload. Fostering knowledge and support in home-based palliative care. Overall, the findings showed that a recently implemented care model improved focus, procedures and collaboration, but also revealed organizational weaknesses. Palliative home care was seen as a nuanced, nonlinear process shaped by patient and family wishes, and end-of-life meetings with patients and families were described as complex, requiring both skilled care and emotional support. Yet some nurses felt insecure and called for more colleagues with whom they could share their experiences, knowledge, and responsibilities.

**Conclusion:**

Supported by a newly implemented structured care model, the home care services worked systematically and purposefully to improve the quality of care for patients with palliative needs. However, the data revealed weaknesses in the system, such as a lack of clear reporting guidelines and unclear roles among collaborating healthcare services. Healthcare workers were engaged and described their work as complex and unpredictable, thus underlining the need for confidence, competence, and support from colleagues. Overall, the study demonstrates that palliative home care is enhanced by clearly articulated goals and a comprehensive understanding of the requirements needed to deliver high-quality care.

**Supplementary Information:**

The online version contains supplementary material available at 10.1186/s12904-026-02121-0.

## Background

There is growing recognition that individuals of all ages with life-limiting conditions may benefit from palliative care, which encompasses the management of physical symptoms alongside focus on emotional, social and existential needs to improve quality of life [1].

To ensure equitable access to palliative care services and dignity at the end of life, the World Health Organization [2] acknowledges palliative care as a core component of universal health coverage and a fundamental human right. WHO member states are being urged to integrate palliative care across all levels of the health system, including primary care and home-based services. The OECD report, Time for Better Care at the End of Life [3], highlights that while most people prefer to die at home, the majority still die in hospital. This is supported by recent research showing that individuals who die at home in high-income countries remains low, and the potential for extending time spent at home and facilitating home deaths is underutilized [4]. The OECD report calls for policy reforms that reduce unnecessary hospitalizations and enable care to be delivered in the setting patients value most – their homes. The EAPC Atlas 2025 [5] reinforces these positions by documenting that 76% of European countries include palliative care in their primary care services, and many have national strategies or laws supporting it. The document recommends expanding service availability with a focus on home-based and community-integrated models.

In-home palliative care models have shown positive benefits, particularly in patient outcomes [6]. This includes improved symptoms, enhanced quality of life and greater alignment with patient preferences [7–10]. A review by Kim and Tarn [7] found that individuals who received care from primary care providers were less likely to be admitted to hospitals or emergency departments, and more likely to be discharged to, or die in, supportive care settings such as a home or a hospice. This is supported by Hui and Bruera [8], who demonstrated that early integration of home-based palliative care significantly increased the number of deaths that occurred at home. Home-based palliative care has also been found to offer substantial cost savings by reducing hospital admissions, intensive care utilization and duration of stay [9]. Seow and Bainbridge [11] underscore that effective home-based palliative care relies on integrated teamwork, compassionate professionals and holistic care. Healthcare workers usually find providing palliative care at home rewarding as it fosters meaningful relationships and personal fulfilment [12]. However, conflicts between their personal beliefs and professional experiences can result in burnout and reduced quality of care, especially when there is a discrepancy between care demands and available resources.

Patients in need of palliative care often require a combination of primary and specialist healthcare services. In Norway, where the present study was conducted, healthcare services are divided between the state, which is responsible for specialist care such as hospital services, and the municipalities which provide primary healthcare services, including general practitioners (GPs), nursing homes, and home care services [13]. Access to primary healthcare services is coordinated through municipal allocation offices. The overarching goal of healthcare delivery is to ensure that patients who want to stay at home are able to and can spend as much time there as possible. Thus, even when patients receive primary home care services, – a specialist palliative care team from the specialist healthcare service, comprising professionals with advanced expertise (e.g., physicians, nurses, physiotherapists etc.) – is involved in patient care. At the same time, all municipal healthcare services are obligated to ensure high quality care, including fostering skills development in palliative care [14, 15].

Nurses and nursing assistants are frontline care providers in home-based palliative care. Through their daily practice, they enact the principles and recommendations embedded in palliative care models and national policies aimed at ensuring high-quality care. Understanding their experiences, particularly the opportunities and challenges they face, can help achieve the overarching goals of enabling patients with palliative care needs to remain at home and die at home, if that is their preference.

## Methods

### Study aim and research question

How do home care services in a Norwegian municipality implement practices that enable patients to die at home, and how do healthcare workers experience these practices?

### Design

This study used a qualitative research design with a thematic secondary analysis [16]. A qualitative approach was chosen to explore the experiences and perspectives of healthcare workers, managers and model developers of the practice of enabling patients to die at home. A person-centered approach with a holistic perspective for both patients and relatives informed the conduct of the study [17].

### Study context

**Fig. 1:**
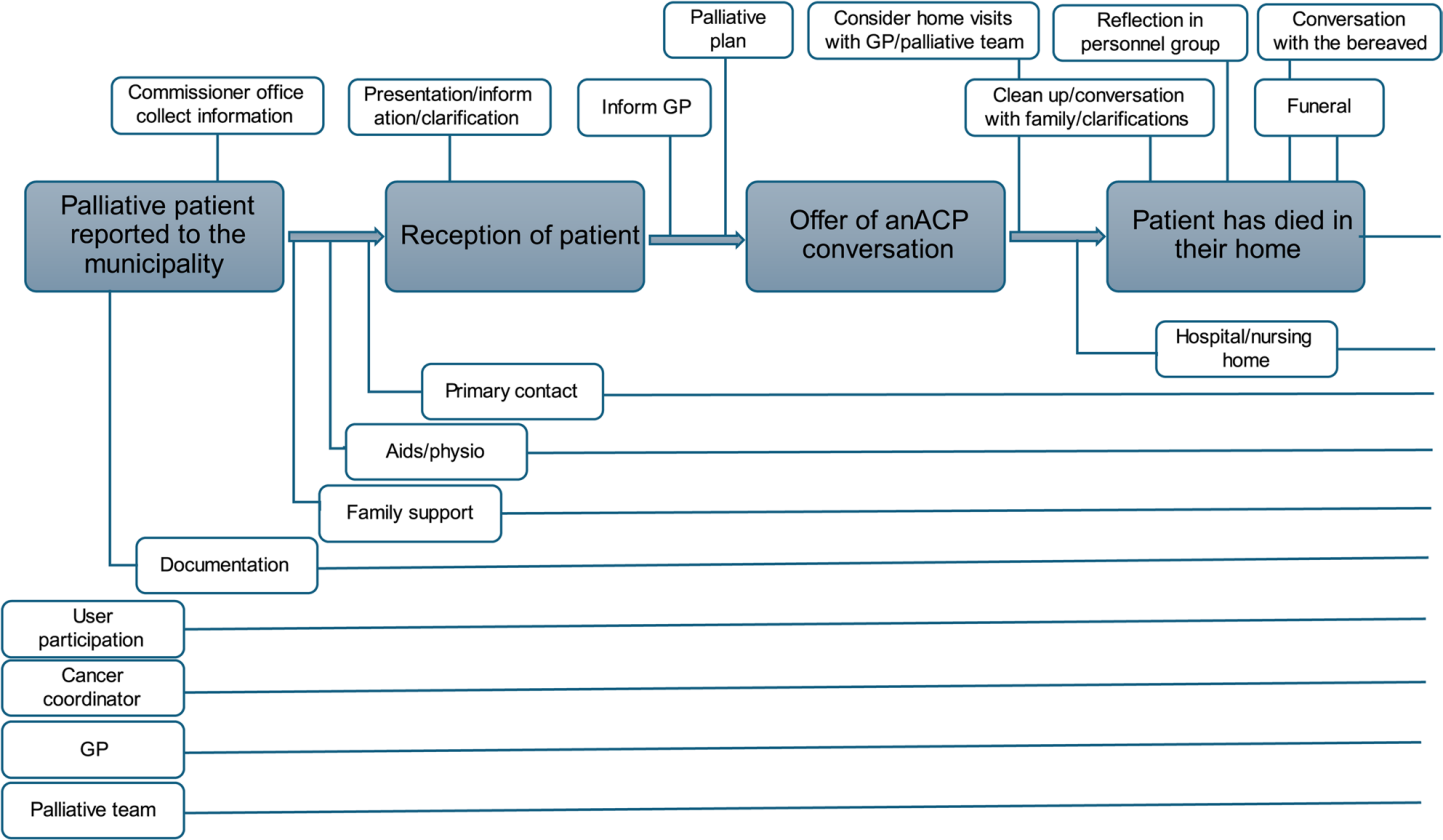
Simplified example of the structured care model designed for patients with palliative care needs

The study was conducted in a medium-sized Norwegian municipality comprising approximately 40,000 inhabitants. The home care services in the included municipality had recently (2022–2024) developed and implemented an innovative and structured care model for days at home and home death (Fig. 1.). A multidisciplinary group of municipal employees and volunteers, appointed by the municipality’s head of care services, collaborated to develop the model by examining challenges related to providing palliative care at home, particularly for patients wishing to die at home. The group, comprising a municipal cancer care coordinator (project manager), home care practitioners and managers, staff from the allocation office, an employee in the municipal palliative care unit, a volunteer, and a patient with palliative cancer, reviewed the entire care pathway from the first notification of need to the period after death. Throughout this process, they identified key challenges and developed solutions to support patients, relatives and healthcare providers throughout the end-of-life journey. The resulting model, conceptualized as a patient pathway tailored to community healthcare services, was designed to strengthen the quality of palliative care by helping patients and families achieve a safe and comforting home environment, including the possibility of dying at home. By visualizing important elements of the palliative care trajectory, the model supports healthcare workers in anticipating potential challenges, staying proactive, and developing individualized palliative care plans. The model was implemented when the data collection of this study was conducted and, to the best of our knowledge, is still being actively used in the included home care services.

### Data collection and characteristics of the participants

Prior to recruitment, approval to conduct the study was obtained from the municipal head of health and care services. Three home care service units, compromising nine sub-units in a single Norwegian municipality, were subsequently recruited. The participants included members of the project group that had developed the structured care model, home care unit managers, and healthcare workers employed in the participating units (Table 1).

**Table 1:** Overview of Participants

Focus groups	Role	Work experience/education	Number of participants
Project Group	Healthcare workers	Oncology/palliative care nurses Assistant nurses with specialized training in palliative care	4
	Volunteers and patient representatives	Previous assistant nurses with specialized training in palliative care Experience of home care	2
	Care coordinators working in the allocation office	Occupational therapists Oncology nurses	2
Home care unit managers (M) from nine units	Unit managers	10–35 years of experience as nurses/managers	9
Healthcare worker (HW) from one unit	Nurses and assistant nurses	Assistant nurses with specialized training in palliative care. Nurses with advanced training in wound care. Nurses with advanced training in ageing, health and society. Registered nurses 1–12 years of experience	5
Healthcare workers from mixed units (HWM)	Nurses and nurse assistants	Palliative care resource nurses Registered nurses Assistant nurses Nurses with specialized training in palliative care 4–35 years of experience	8
Total			30

Members of the project group were invited to participate in a focus group [18] by the project manager. The group comprised two home care unit managers, four healthcare workers, one volunteer, one patient representative and two officers from the allocation office. Eight out of ten group members agreed to take part in the focus group.

To explore the organizational aspects of palliative care and the facilitation of home deaths, unit managers were also invited to a separate focus group. Nine home care unit managers, one from each unit that had adopted the structured care model, agreed to participate.

Healthcare workers, including nurses and assistant nurses from the relevant units, were recruited to participate in the focus groups through their managers. Two focus groups were conducted, one comprising five participants from a single home care unit, and the other comprising eight participants representing several home care units. An overview of all the participants is provided in Table 1.

All the participants were woman, and they represented a wide range of professional experience from newly educated staff to individuals with more than 30 years of practice. The focus groups took place in dedicated facilities outside the home care units in May 2024. KV and KÅE conducted the focus groups with the project group, while the managers MKG and IE conducted the focus groups with the healthcare workers. There was rotation between the roles of moderator and assistant moderator in the different sessions.

The average duration of the focus group interviews was 71. 25 min (interview guides are available in supplementary file 1). All focus group interviews were audio recorded.

### Data analysis

This study is based on a secondary analysis [16] of qualitative data originally collected for an evaluation report for the structured care model for days at home and home death, commissioned by The Norwegian Directorate of Health (not cited to protect the anonymity of the participants). Verbatim transcriptions of the focus group recordings were conducted by KV (managers), HH (project group) and IE and MKG (healthcare workers).

All authors contributed to the analysis of the initial report, as well as the re-analysis, and all previously collected data were re-analyzed [16] between May and August 2025. As the initial report, the secondary analysis adopted Braun and Clarke’s [19] thematic approach to explore new dimensions of the dataset in light of a revised research question. This approach was chosen to ensure methodological consistency and because it allows for a deeper, interpretive engagement with the data. Braun and Clarke’s thematic analysis is particularly well suited to focus group material, where meaning is co-constructed through participant interaction and where interpretive flexibility is needed to capture complexity across perspectives. The method supports an iterative, researcher-generated development of themes, which was essential for exploring new layers of meaning within the existing dataset and for generating conceptually rich themes that not only describe but also provide a more analytical understanding of the phenomenon under study [19]. In line with the first phase of thematic analysis, the analysis process began with renewed familiarization with the data in light of the new focus [19]. Each author (KV, HH, IE, KÅE, MKG) conducted the initial review independently. Authors then convened to discuss and compare their interpretations, allowing for collaborative exploration of differing perspectives in the data set. Next, two authors (MKG and IE) independently coded the data. Prior to a joint meeting with the full author group, MKG and IE compared their coding to identify differences or discrepancies. These initial codes were then discussed and refined collaboratively in the author group. Based on this process, preliminary themes were developed and subsequently refined through several rounds of analysis meetings. An overview of the identified themes and associated codes is provided in supplementary file 2.

## Findings

The analyses resulted in the following three themes describing how home care services implement practices enabling patients to die at home, as well as their experiences of this practice: T1) Advancing palliative care through focused projects: enhancing competence and addressing challenges, T2) Spending the final days at home: identifying patients with palliative care needs and providing compassionate and effective care, and T3) Sharing responsibility versus reducing the workload: Fostering knowledge and support in home-based palliative care. The results are presented according to the identified themes below.

### T1: advancing palliative care through focused projects: enhancing competence and addressing challenges

Across all four focus groups, participants described how the recent implementation of a structured care model increased the focus of healthcare workers on palliative care and days at home. They stated that their procedures had improved, that they were more proactive and structured in their work, and that collaboration with other healthcare workers had become more effective.

“I feel that we’ve become a lot more… well, yeah… we kind of knew what we were supposed to do before as well, but I feel like – even though we don’t actually use it [in practice] – I still think we’ve become much more routine based because we’ve gone over the model several times, so even if we don’t actively use it when we get new patients, it’s still helped us improve our procedures. Also, it [the model] may have contributed to better communication with the allocation office, like the office has got better at collecting information, which was not the case before” (Healthcare worker 1, focus group 2).

Furthermore, both healthcare workers and managers spoke favorably of the model for serving as a reliable support system, helping to ensure that all steps in the care process were remembered. This contributed to a stronger sense of security and organization in care delivery, while also enhancing the quality of care and improving efficiency. One manager stated that her employees had become more self-reliant after the implementation of the model, and that healthcare workers had become better at evaluating and reflecting over the care they provided to this patient group, without always involving them.

However, the implementation of the structured care model also exposed organizational shortcomings in palliative home care and home deaths in the home care units and across collaborating healthcare workers for example, during the model development phase, the project group discovered that many general practitioners (GPs) were unclear about their responsibilities concerning patients with palliative care needs receiving home care. There was confusion over the roles of hospital physicians, the physicians on the hospital’s palliative care team, and GPs, particularly regarding patient communication, symptom management and prescriptions. This discovery was supported by the healthcare workers, who had experienced problems in obtaining the necessary prescriptions and /or reaching the respective GP and/or palliative care team when needed, particularly during weekends.

Another aspect discussed by healthcare workers during the focus groups was a lack of clarity in how and where to document patient care in cases of palliative home care and home death. This was particularly evident in relation to ACP conversations, which involve structured discussions with patients about their preferences for future care.

In both focus groups, healthcare workers talked about where such aspects of care should be documented, and what the best solution would be:

“I was thinking about the documentation and that it could be placed in the palliative care plan… Yes, it was… it was [supposed to be] under assessment or information [in the electronic documentation program]. I don’t remember exactly (…) but that’s where we document ACP conversations (…)” (Healthcare worker 5, focus group 2).

As the discussion continued, it became evident that not all healthcare workers were aware of how or where APC conversations should be documented. For example, some healthcare workers knew about a recently added location for documenting these conversations, while others stated that they had neither heard of nor seen it.

The ACP conversations themselves also appeared to present challenges for the home care teams, more specifically, to separate structured ACP conversations in line with the national guidelines, and the more informal conversations about palliative home care and home death. Although the ACP conversations were an established part of the structured care model, in practice it was difficult to keep these conversations separate, which also caused a lack of clarity about where to document the conversations.

“We also conduct ACP conversations without being aware of it, for example, when we talk with them, sit down and ask them what’s important to them. ‘If you get really sick, would you like to go to a nursing home for a while? Or would you prefer to stay at home? (…)’ But are we to document such settings as an ACP conversation?” (Healthcare worker 5, focus group 2).

This was also evident in the focus group discussions about these conversations, where many healthcare workers used the same term (ACP conversation), whether they were talking about formal or informal conversations. This distinction appeared to be more clearly understood by the managers and the project group. Nevertheless, according to the healthcare workers, the model had increased their focus on engaging in conversations about palliative home care and home deaths in general.

### T2: spending the final days at home: identifying patients with palliative care needs and providing compassionate and effective care

When asked about the process of palliative home care and home deaths, the participants were forthcoming in sharing their experiences. This revealed a more nuanced and non-linear trajectory than had been outlined in the structured care model and emphasized the importance of patients and their families’ wishes.

According to the healthcare workers, the process of initiating palliative home care or planning for home death typically began when the home care services received notification of a new patient from the allocation office. If they did not know the patient, they familiarized themselves with the patient’s medical history by going through their records. A “first-time visit” or a “first time conversation”, which some healthcare workers also called a “assessment visit”, was initiated when the patient was transferred to the home care service. The aim was to clarify and assess the patient’s wishes, how much, and what help they needed, clarify what the home care service could offer, assess what aids were needed (e.g., hospital bed, toilet chair) and ensure that the patient was referred to the palliative care team. At the start of the process, the level of care that the patients wanted varied. Some patients just needed a point of contact – a phone number and/or a contact person. Either way, the healthcare workers stated that it was important to start building a relationship with the patients and their families at an early stage and also accommodate their wishes and needs as much as possible.

One healthcare worker shared a story about a home death, illustrating how real-life practice can sometimes differ from standard procedures and how healthcare workers adapt to address changing needs:

“I had planned a conversation. But [in this particular case] it went so fast…. I was there on the Thursday, I think, and made an appointment for an ACP conversation for the following Monday. I also offered him a conversation on the Friday, but he wanted to wait for his family to arrive [on the Monday] as he also wanted them to participate in the conversation. But when I visited him on the Friday he was really ill, and we needed to provide a hospital bed and everything [else needed] on the spot. Then on Monday he died (…). Initially, they were very uncertain about it… whether his wife could handle him dying at home. But we had a lot of conversations [about home death], explaining what the home care services could provide, what we could offer in terms of pain relief and sedatives. So, they changed their minds and decided he would stay at home. His wife said afterwards that she was grateful for this… even if we didn’t follow the plan to the letter… we were still able to get through it (…)” (Healthcare worker 1, focus group 2).

There were ongoing discussions among the participants concerning when, and for whom, to initiate palliative home care and support for home deaths. For example, caseworkers working in the allocation office stated they could better support patients who wished to die at home if they received earlier and more detailed information about their wishes – giving their families time to prepare. Healthcare workers and managers were concerned about how to identify which individuals had palliative care needs. During the discussions, several healthcare workers realized that most of their patients were actually in need of palliative care, even though they had not been formally defined as needing such care. When reflecting on the definition of a patient with palliative care needs, the healthcare workers concluded that this referred to patients who could no longer receive curative treatment and whose care would focus solely on symptom management, as well as patients who they believed would likely die within a year or two. During the discussion, several other patients groups who might fall under this category, but who were not typically offered palliative care, were mentioned, including patients with Parkinson’s disease, renal failure, heart failure, patients receiving dialysis, and patients with dementia. Many healthcare workers noted that this definition could apply to almost all the patients they cared for, but that patients who were clearly identified as having palliative care needs were approached differently:

“I feel that when patients with obvious palliative care needs arrive home, they’re allocated much more time to conduct the first visits, to assess, you know. So, I feel that patients who are clearly allocated this category [defined patient with palliative care needs] are given plenty of time [for visits]. But generally, we often get Parkinson patients, patients with dementia, and we’re not thinking about it in the same way, and the assessment visits are often ‘in and out’ [short and fast]” (Healthcare worker 1, focus group 2).

### T3: sharing responsibility versus reducing the workload: Fostering knowledge and support in home-based palliative care

When patient deaths were anticipated in the near future, participants in all four focus groups described meetings with patients and their relatives as complex, involving the provision of high-quality care and the emotional support of loved ones.

“They [healthcare workers] want to do a good job for the patient and their families. Because they know that it’s a special situation for both themselves and the [the patient’s] relatives. For nurses in these settings, there are many considerations to take into account. They need to stay focused on the patient and make sure that their pain is adequately managed. You become very engaged, right? There are also relatives who are wondering about different things…(…)” (Manager 7).

The participants highlighted confidence as an essential component when working with complex palliative care cases and supporting patients dying at home. As one healthcare worker noted: “You can’t be uncertain in situations like these” (Healthcare worker 3, focus group 2). Nevertheless, a recurring challenge reported by the participants was that some nurses lacked the necessary level of confidence to effectively engage with this patient group. Several nurses emphasized the need for more nurse colleagues to support knowledge sharing, as well as help share the responsibility of managing the complex tasks involved in this kind of patient care, rather than having to manage such tasks on their own.

“Sometimes there are two nurses present [in palliative care situations] because one of them feels insecure, and the other is there to offer support, especially during weekends when nurses have to manage their tasks on their own” (Healthcare worker 4, focus group 2).

The managers also noted that nurses often preferred to have a nurse colleague accompany them when caring for palliative care patients. This was attributed to the infrequency of such encounters and a sense of insecurity experienced by some nurses. At the same time, several of the managers expressed their concern that nurses rarely asked for assistant nurses— despite their experience of palliative care and familiarity with the patient.

“We have assistant nurses with advanced training [in palliative care] on our team, and they simply haven’t been allowed to get involved” (Manager 4).

As reflected in the data material, the competence of healthcare workers could stem from experience, working in the project group that developed the structured care model, or from pursuing continuing education in palliative care. According to the study participants, those healthcare workers who had gained competence through participation in the project or through continuing education would automatically assume greater responsibility for this patient group. These healthcare workers, described as *champions* by the study participant, were making critical contributions to maintaining focus and ensuring quality in palliative care. At the same time, experience indicated that when a *champion* left their position, the increased focus on palliative care in the unit decreased, underlining the potential weakness of relying on such individuals. Nevertheless, all the participants emphasized the importance of having dedicated nurses to sustain competence, as well as building and maintaining focus on quality of palliative care.

Despite not always feeling competent enough to handle tasks related to palliative care and home deaths, several healthcare workers highlighted that they had participated in professional development days (initiated by the unit), as well as courses on the subject. They underlined the need for continuous training and receiving timely information, as well as the opportunity for knowledge exchange among their colleagues. All the participants agreed that increasing competence for all staff was important to ensure that everyone felt confident in conducting both ACP conversations and other care tasks related to palliative care, and that this should be initiated and funded by the municipality. Also, both healthcare workers and managers emphasized the importance of reflection among colleagues following a home death. Healthcare workers described a need for more time to engage in discussions before, during and after such events. Managers noted an increased need for reflection among healthcare workers and believed that opportunities for reflection had improved over time. They also highlighted the importance of fostering such discussions.

## Discussion

Home-based palliative care plays a critical role in enhancing the quality of life for patients facing life-limiting illnesses. The study’s findings describe how the home care services in a Norwegian municipality implemented practices to enable patients to die at home, and how healthcare workers experienced these practices.

Overall, the findings suggest that implementing a structured care model for days at home and home death enabled the home care services to work more systematically and purposefully, improving the quality of care for this patient group. The model clarified service delivery goals, highlighted key elements of high-quality care, and revealed potential challenges. The model also appeared to enhance healthcare workers’ sense of security, streamline care provision, and improve the efficiency of palliative care. Although healthcare workers reported using the model in practice, they did not typically follow it in a step-by-step manner but rather viewed it as a reminder of their existing practice.

### Identifying patients with palliative care needs

According to the findings, palliative home care or planning for home death typically started when the home care services were notified about a new patient, often a cancer patient, being discharged from hospital. However, the data material revealed uncertainty surrounding the definition of a patient with palliative care needs.

During the focus group discussions on this topic, healthcare workers recognized the extent to which they were already providing care for patients with gradually increasing palliative care needs, and that this definition encompassed a broader range of patients than patients diagnosed with cancer. This made them question whether this broader patient group should be included in the structured care model and their overall palliative care work. This is supported by Radbruch et al. [1], who underline that palliative care should be available to all people living with serious health-related suffering, regardless of diagnosis, prognosis, age or setting. As described in the current study, the patient population in home-based care largely comprises individuals with complex, chronic and life-threatening conditions. Identifying patients in need of palliative care is essential, as the early initiation of home-based palliative care is linked to better symptom control and increased likelihood of patients dying at home [8]. The supportive and palliative care indicators tool (SPICT™) can help identify patients with palliative care needs [20]. According to the project group of the structured care model in the present study, this tool had been introduced to the healthcare workers as part of their training for the model. However, none of the healthcare workers referred to SPICT as a tool they used in their daily practice, suggesting that additional support and training may have been needed to strengthen its integration during implementation [21].

When the healthcare workers discussed patients with palliative care needs who might benefit from palliative home care or a planned home death, it became apparent that they were often actually describing end-of-life care. They often used terms like “alleviating symptoms” or “palliative care”, which are related but distinct [22]. *Alleviation* refers to reducing suffering in both curative and palliative contexts, while end-of-life care targets the final phase of life [22]. Palliative care, however, is recommended from diagnosis until death [1]. Conceptual ambiguity in clinical practice can delay palliative care and hinder communication among healthcare workers. Thus, emphasizing how different terminology is understood is recommended when implementing palliative care models in the workplace. The precise and consistent use of terminology can facilitate earlier integration of palliative care services and enhance interdisciplinary collaboration [1]. Moreover, the terminology used is of critical importance when communicating with patients and their families, as it shapes their understanding of the care trajectory, expectations and available support [23].

### Planning for increased time at home and home death

A positive outcome of implementing the care model was an increased focus on having conversations about palliative home care and home deaths with patients and their relatives. However, even though ACP conversations were defined as part of the structured care model, are considered essential for providing high-quality palliative care [24], and are supported by national implementation guidelines [25], these conversations still posed challenges for the healthcare workers. They had difficulties in distinguishing between a planned ACP conversation, defined as a process that enables individuals to define goals and preferences for future medical treatment and care, to discuss these with family and healthcare providers, and to record and review these preferences if appropriate ([26] p. 544) and more informal talks about palliative care and home deaths. This lead to uncertainty about documentation.

The national recommendations for ACP are relatively new in Norway, which may lead to unclear usage of the term and a lack of understanding of what ACP conversations entail. Ideally, such conversations are planned in advance, giving patients the opportunity to involve their loved ones as they choose. The planning should be carried out by an interdisciplinary team, with follow-up conversations turning points in the disease trajectory [25, 26]. Consequently, there is a significant difference between these formal discussions and those discussions that happen spontaneously around the kitchen table. When healthcare workers label both kinds of conversations ACP, and are uncertain about how to document them, it could be questioned whether this reflects a lack of competence or indicates a gap between the guidelines and what is practically achievable in everyday work. A scoping review by Wilkin et al. [27] identified lack of confidence, competence, role ambiguity and prognostic uncertainty as key barriers for implementing ACP conversations. The findings of this study underscore the challenge of identifying the appropriate time to initiate formal ACP conversations. However, the model had increased the focus of healthcare workers on having conversations about palliative home care in general with their patients, meaning these conversations can serve as a foundation for more structured advance care planning as they help patients feel emotionally prepared and more open to engage in formal discussions with the interdisciplinary team [28]. This suggests that informal dialogue is not merely supplementary, but may play a critical role in facilitating meaningful and timely formal planning in palliative care, thereby indicating that healthcare workers should strive to ensure a balance between informal and formal conversations.

### Responsibility and support

The data material revealed the challenges faced by home care services in supporting extended stays at home and enabling dying at home, especially when patients or relatives were hesitant about accepting healthcare support. This reluctance was sometimes related to concerns about too much medical equipment in the home or the differing needs between patients and families. Fringer et al. [29] found that a key phenomenon observed among palliative care patients and their families was maintaining a sense of normality, and that the transitions in a care trajectory often took place subconsciously when the crisis occurred and responsive actions were necessary. This might explain why healthcare workers struggle to establish proactive support rather than waiting for a crisis. The results of the study also demonstrate how quickly situations can change over the course of a weekend. Thus, timely care and preparedness among patients and families are crucial for home-based palliative care [11, 30]. Establishing routine practices is important, since receiving more comprehensive palliative care can improve symptoms, enhance quality of life and reduce hospitalization [7, 10].

The data material offers valuable insights into how healthcare workers and managers experience the responsibility of providing compassionate and effective palliative care. Managing rapidly evolving and complex care situations, while facilitating extended time at home and for home death, presents both professional and emotional challenges. In line with this, the healthcare workers in the current study reported emotional strain when caring for dying patients and their families, including stress, emotional fatigue and a need for support. The ability to develop experience and maintain continuity, which is important for providing high-quality care, was often limited due to long intervals between cases. Despite these challenges – and consistent with the findings of Wu et al. [31], and Ervik et al. [32] – healthcare workers showed a strong commitment to providing high-quality care and worked hard to honor patients’ wishes to die at home, often going to great lengths to enable this.

An interesting finding was, however, how healthcare workers and managers perceived the distribution of responsibilities between nurses and healthcare assistants in such situations. The nurses emphasized the need to collaborate with fellow nurses and to share responsibility with someone of equivalent competence when managing complex palliative care situations, especially in terminal care. Managers also noted that nurses often preferred to work alongside a fellow nurse in palliative situations yet sought to promote a shift toward involving and utilizing all available resources. The managers’ viewpoints are substantiated by and in line with the growing challenges associated with shortages in the global healthcare workforce [33]. However, the nurses’ perspectives should be taken into account as they carry the broader responsibility, and the ultimate accountability.

Peer collaboration has been shown to foster shared responsibility and contribute to a sense of professional self-efficacy in complex palliative care situations [34]. The nurses in this study described a need for confidence when managing palliative care situations, yet many of them lacked professional self-efficacy, thereby indicating a need for increased peer collaboration in this context [34]. Nevertheless, in line with the findings of Forward et al. [35], healthcare workers often manage complex and distressing situations and navigate their own emotions, as well as those of their patients and relatives.

Tan et al. [12] found that continuous support is crucial for healthcare workers to deliver compassionate, high-quality care. In the studied municipality, staff reflection was institutionalized through a care model that promoted debriefing and knowledge sharing among professionals involved in home deaths. However, a distinction emerged: managers believed that they adequately facilitated reflection, while healthcare workers desired more time for reflection before, during, and after patient situations. On the one hand, this may indicate that healthcare workers struggle to build confidence and manage emotional responses due to the infrequency of such situations. On the other hand, it may reflect a mismatch between managers’ perceptions of staff needs and their actual needs. Nevertheless, the nurses in Spelten et al. [36] highlighted the emotional toll of their work and the importance of debriefing. The findings of the present study suggest that having procedures for post-event reflection is insufficient; targeted training and preparation are also needed. Peer support from peers and shared learning opportunities, along with fostering self-awareness and emotional resilience, can enhance the quality of care and ongoing professional development [28]. In this study, champions were described as a valuable asset in terms of both professional and emotional support. These nurses are described as experts who see solutions rather than problems, willingly help others, and take the initiative to make improvements [37]. In the sensitive and complex field of palliative care, these individuals serve as role models, demonstrate good practice and clearly communicate what needs to be prioritized to provide effective palliative care [38, 39]. Their passion and commitment seem to inspire the healthcare workers. However, in this study, the potential departure of these nurses was described as a vulnerability in the system. This emphasizes the vital role of managers in strengthening the skills of all staff to boost their confidence in providing palliative care.

Midlöv and Lindberg [40] emphasize that district nurses require advanced skills in palliative care. Similar to the current study, research highlights gaps in training related to patient assessment, symptom management, communication and end-of-life preparation [31, 41]. Kim et al. [42] also identified a specific need for competence development among home care nurses caring for non-cancer patients, a need that is also evident in the current study. Addressing these gaps requires structured internal training, including realistic simulation scenarios, to strengthen clinical skills and foster collaborative reflection. Managerial support through adequate resources, training and organizational frameworks is essential to ensure sustainable home-based palliative care.

## Strengths and limitations

The data material from this study was analyzed using a secondary analysis approach. While this may result in some overlap with the findings from the initial report, a distinct research question was formulated for the secondary analysis, and a comprehensive independent analytical process was conducted. This led to the identification of new dimensions in the data material. A general limitation of a secondary analysis [16] is the potential lack of contextual familiarity with the original data collection, as well as ethical concerns related to participant anonymity. In this study, however, both the data collection and the subsequent analysis – original and secondary – were conducted by the same research team, thereby mitigating these concerns. Moreover, a secondary analysis of the data material was planned ahead of the data collection. This enabled the research group to take the necessary precautions during the development of the interview guides (e.g., including questions beyond the experiences connected to the implemented care model).

The recruitment of healthcare workers was carried out by their line manager This may have led to a perceived pressure to participate in the study [43]. To mitigate this potential influence, the participants were specifically informed of their right to withdraw from the study prior to the start of the interviews. Moreover, one of the authors took on the dual role as both researcher and participant in the project group. To safeguard the anonymity of the other participants, this participant was not granted access to any data containing personal information from the other focus groups. While this dual role offered unique insights into the context of the recruited home care services during the analysis, it also required particular care to ensure that their involvement did not influence the interpretation of the data [44]. To limit any potential influence on the findings, this author did not participate in writing the results section. Reflexivity during the analysis was addressed through strict adherence to the interview data. This was clarified in the author group ahead of data collection, where openness about eventual issues or disagreements was agreed upon. Lastly, the two focus groups comprising healthcare workers had different compositions. One focus group comprised healthcare workers from one home care unit, while the other group comprised healthcare workers from mixed home care services. The group comprising participants from a single unit that shared procedures and organizational context appeared to facilitate more rapid agreement and less critical negotiation of viewpoints, whereas the group comprising participants from mixed units engaged in more comparisons, debates and clarifications, which seemed to generate more nuanced reflections. This could have influenced the findings and should be taken into account.

## Further research

This study focused exclusively on the perspectives of healthcare workers. To gain a broader understanding, further research should explore patients’ own experiences, preferences and challenges of being at home toward the end of life [45], as well as to understand which services are truly essential for the family to enable patients to remain at home [30]. Moreover, quantitative studies can help identify which care models lead to the best quality outcomes. For example, in the current municipality, a full scale implementation of the model through a complex intervention framework [46] could have contributed to a better understanding of the model’s feasibility, as well as provided a full scale evaluation of the models usability, effect and impact.

## Conclusion

The findings of this study indicate that a newly implemented care model designed to promote days at home and home death enabled home care services to undertake systematic, goal-oriented initiatives to improve the quality of palliative care. The data revealed both strengths and weaknesses in existing practices related to patient and family relationships, collaboration among care staff, service organization, as well as cooperation with external agencies. The service is enhanced by clearly articulated goals and a comprehensive understanding of the requirements needed to deliver high-quality care. To promote increased days at home and facilitate home deaths, it is essential to identify patients with palliative care needs and provide compassionate and effective care. Planning for enhanced days at home and end-of-life care includes conducting both informal and formal advance care planning (ACP) conversations. Delivering high-quality palliative care presents challenges for healthcare workers. However, shared responsibility and peer support contribute to knowledge development in the field of palliative care.

## Supplementary Information

Supplementary Material 1.

Supplementary Material 2.

## Data Availability

The datasets used or analyzed in this study are available from the corresponding author upon reasonable request.

## Abbreviations

ACP: Advance care planning
GPs: General practitioners
