# Supplementary material for: Practices and perspectives on dying at home in Norwegian home care services – a secondary analysis of qualitative data
Source: BMC Palliat Care. 2026 May 1;25:176. doi: 10.1186/s12904-026-02121-0 (PMC13276947; doi:10.1186/s12904-026-02121-0)
Supplement: Supplementary file 1 — Supplementary Material 1. [file 12904_2026_2121_MOESM1_ESM.docx]

**Focus Group Interview with the Project Group in the HEIM Project – Home Time/Home Death**

At the start, information is provided about the purpose of the focus group interview, the timeframe, and that data will be anonymized. It is explained that a focus group is a structured conversation where participants can speak freely, but the moderator may interrupt. It is emphasized that we are not looking for “correct answers” or consensus, but rather a variety of viewpoints, with the conversation flowing between group participants rather than only responding to the moderator. There will be an opportunity to ask clarification questions.

**Topic Guide**

**Roundtable introductions so everyone can present themselves:**
Can you tell us a little about your role/position and how long you have held this role/worked in the municipality?

**Theme: Developing the Model**

- How did you work to develop the model for increased home time and home death?
  (Professional inspiration, inspiration from other municipalities, inspiration from other models, own experience or the municipality’s existing services).

Collaboration within the project group.
Were your viewpoints heard and included?

- How did you go about including collaboration between different actors when developing the model?
  Patient pathways, specialist health services, general practitioners, interdisciplinary collaboration, relatives, volunteers, and cancer coordinator.

**Theme: Implementing the Model**

- What are your thoughts on the municipality implementing a model for increased home time and home death?
- How did you proceed to implement the model in the municipality?
  (Oral/written information, resource persons, ensuring it is used)

**Theme: Evaluating the Model**

- What do you think about the finalized model?
  Its relevance for practice.
  Is it being used? What is needed for it to be used? What hinders its use?

**Theme: Quality in Services**

- Do you have any thoughts on whether the model contributes to safety and security for patients and relatives?
  Strengths/weaknesses
- Do you have any thoughts on whether the model contributes to dignity for patients and relatives?
  Strengths/weaknesses
- Has the model made you more aware of or changed your attitudes toward increased home time and home death for patients in the palliative phase? If so, how?
- Do you have suggestions for possible areas of improvement for the model?
- Are there other things you would like to share about the development and implementation of the model?
