# Supplementary material for: Practices and perspectives on dying at home in Norwegian home care services – a secondary analysis of qualitative data
Source: BMC Palliat Care. 2026 May 1;25:176. doi: 10.1186/s12904-026-02121-0 (PMC13276947; doi:10.1186/s12904-026-02121-0)
Supplement: Supplementary file 2 — Supplementary Material 2. [file 12904_2026_2121_MOESM2_ESM.docx]

| Themes | Codes |
| --- | --- |
| T1: Advancing palliative care through focused projects: enhancing competence and addressing challenge | More skilled in palliative care and facilitating home deaths across all levels of the healthcare system |
|  | Better cooperation across healthcare services |
|  | Prescriptions (for medication) and contact with the GP can be challenging |
|  | Unclear division of roles and responsibilities among collaborating physicians |
|  | A newly implemented structured care model has increased focus and is functioning as an aid |
|  | Unclarity in terms of documentation when working with *patients with palliative care needs* and home deaths |
|  | There has been an ongoing capacity building within palliative care and home deaths until now |
| T2: Spending the final days at home: identifying patients with palliative care needs and providing compassionate and effective care | Lack of knowledge among patients, relatives, and hospitals about the home‑care services related to days at home and home death |
|  | The process of planned days at home and home death in practice |
|  | The process of planning days at home/home death should start earlier |
|  | Unclear distinction between formal and informal advance care conversations in practice |
|  | Uncertainty regarding the routine for formal and informal advance care conversations |
|  | The valuable, spontaneous conversations |
|  | Who is actually considered a patient with palliative care needs? |
|  | The importance of having a relationship with the patient |
|  | Not all patients and/or relatives are open to conversations about death |
|  | Working to normalize and reduce the discomfort around conversations about death |
|  | Managing differing wishes between the patient and relatives while supporting both parties can be challenging |
|  | Healthcare personnel and relatives often have positive experiences with home death |
|  | Not everyone can, wants to, or should die at home |
|  | The wishes of the patient and/or relatives are highly prioritized |
| T3: Sharing responsibility versus taking the load of: Fostering knowledge and support in home based palliative care | Not all healthcare personnel feel confident working with this patient group |
|  | A need for increased knowledge among all healthcare personnel, not only nurses |
|  | Healthcare personnel need continuous training and information about days at home/home death |
|  | Healthcare personnel draw on each other’s knowledge across departments |
|  | Healthcare personnel support each other and maintain openness about difficult situations |
|  | Dependence on key drivers to maintain continuous focus on and work with time at home/home death |
|  | Healthcare personnel need time for reflection before, during, and after time at home/home death |
